# Effect of Parenteral Selenium Supplementation in Critically Ill Patients: A Systematic Review and Meta-Analysis

**DOI:** 10.1371/journal.pone.0054431

**Published:** 2013-01-25

**Authors:** Ting-Shuo Huang, Yu-Chiau Shyu, Huang-Yang Chen, Li-Mei Lin, Chia-Ying Lo, Shin-Sheng Yuan, Pei-Jer Chen

**Affiliations:** 1 Division of General Surgery, Department of Surgery, Chang Gung Memorial Hospital, Keelung Branch, Keelung, Taiwan; 2 Institute of Biopharmaceutical Sciences, National Yang-Ming University, Taipei, Taiwan; 3 Department of Education and Research, Taipei City Hospital, Taipei, Taiwan; 4 Department of Pharmacy, Chang Gung Memorial Hospital, Keelung Branch, Keelung, Taiwan; 5 Institute of Statistical Science, Academia Sinica, Taipei, Taiwan; 6 Graduate Institute of Clinical Medicine, National Taiwan University College of Medicine, Taipei, Taiwan; Charité University Medicine Berlin, Germany

## Abstract

**Background:**

It is currently unclear whether parenteral selenium supplementation should be recommended in the management of critically ill patients. Here we conducted a systematic review and meta-analysis to assess the efficacy of parenteral selenium supplementation on clinical outcomes.

**Methods/Principal Findings:**

Randomized trials investigating parenteral selenium supplementation administered in addition to standard of care to critically ill patients were included. CENTRAL, Medline, EMBASE, the Science Citation Index, and CINAHL were searched with complementary manual searches. The primary outcome was all-cause mortality. Trials published in any language were included. Two authors independently extracted data and assessed trial quality. A third author was consulted to resolve disagreements and for quality assurance. Twelve trials were included and meta-analysis was performed on nine trials that recruited critically ill septic patients. These comprised 965 participants in total. Of these, 148 patients (30.7%) in the treatment groups, and 180 patients (37.3%) in control groups died. Parenteral selenium treatment significantly reduced all-cause mortality in critically ill patients with sepsis (relative risk [RR] 0.83, 95% CI 0.70–0.99, *p* = 0.04, *I^2^* = 0%). Subgroup analyses demonstrated that the administration schedule employing longer duration (RR 0.77, 95% CI 0.63–0.94, *p* = 0.01, *I^2^* = 0%), loading boluses (RR 0.73, 95% CI 0.58–0.94, *p* = 0.01, *I^2^* = 0%) or high-dose selenium treatment (RR 0.77, 95% CI 0.61–0.99, *p* = 0.04, *I^2^* = 0%) might be associated with a lower mortality risk. There was no evidence of adverse events.

**Conclusions/Significance:**

Parenteral selenium supplementation reduces risk of mortality among critically ill patients with sepsis. Owing to the varied methodological quality of the studies, future high-quality randomized trials that directly focus on the effect of adequate-duration of parenteral selenium supplementation for severe septic patients are needed to confirm our results. Clinicians should consider these findings when treating this high-risk population.

**Systematic Review Registration:**

PROSPERO 2011; CRD42011001768

## Introduction

Sepsis is the leading cause of mortality in critically ill patients and its incidence has increased over the past few decades [1]–[4]. Even with early diagnosis, proper antibiotics, and evidence-based supportive care, mortality rates associated with severe sepsis or septic shock remain high [5], [6]. An up-to-date systematic review demonstrated that mortality rates among general intensive care unit (ICU) patients with sepsis and septic shock ranges from 21% to 53% [1]. Although noteworthy scientific advances have provided new insights into the pathophysiology of severe sepsis and septic shock, there has been difficulty translating novel therapies into clinical practice [4], [5]. Recently, pharmaconutrients have shown the potential to improve clinical outcomes through pharmacologic modulation of systemic inflammation and immune response when delivered at supraphysiological doses to cells involved in response to injury or illness [7]–[9].

Selenium is an essential micronutrient crucially important to human health. Selenium is the only trace element to be specified in the genetic code, as selenocysteine, a component of selenoproteins. Selenocysteine is inserted into the active centre of functional pivotal selenoproteins and has a range of pleiotropic effects, including antioxidant and immunomodulatory, and increasing anti-viral immunity [10], [11]. Among these selenoproteins, the glutathione peroxidase (GPx) selenoenzymes (GPx-1 and GPx-3) and selenoprotein P, play a role in protecting cells from free radical-induced oxidative stress [10]. Almost all patients with sepsis admitted to ICUs have low plasma selenium levels and GPx-3 activity, which correlates inversely with severity of sepsis and mortality rate [12], [13]. Moreover, low plasma selenium levels are associated with an increasing risk of nosocomial infections [12].

It is currently unclear whether parenteral selenium supplementation should be routinely administered to critically ill patients with sepsis [14], [15]. Systematic reviews suggest that there is insufficient evidence to recommend parenteral selenium supplementation in critically ill adults [16]. Randomized trials involving parenteral selenium supplementation in critically ill patients with sepsis have yielded contradictory results [17]–[19]. In addition, in heterogeneous intensive care populations with a broad spectrum of diseases, the use of mixed nutrients and the lack of an optimal administration schedule (i.e., dose, route, timing, and duration) compromise interpretation of the results of clinical trials. In this study, we performed a systematic review of randomized trials involving parenteral selenium supplementation in critically ill patients and a meta-analysis of critically ill patients with systemic inflammatory response syndrome (SIRS) or sepsis to assist practitioners/researchers in appropriately determining the efficacy of such a strategy.

## Materials and Methods

### Search strategy

This review was conducted prospectively according to our published protocol that included analysis planning [20]. Searches were not restricted by language, publication status, or date. Search terms used were ‘selenium’ and ‘selenium compounds’. We combined exploration of MeSH headings using a truncation strategy (selen*), and applying the filter method for randomized controlled trials as suggested in the Cochrane Handbook for Systematic Reviews of Interventions (http://www.cochrane-handbook.org) to narrow down the number of articles. We searched the following databases: CENTRAL (the Cochrane Library, latest issue December 2011), MEDLINE (January 1950 to December 2011), EMBASE (January 1980 to December 2011), CINAHL (January 1982 to December 2011), and the Science Citation Index (January 1981 to December 2011). The MEDLINE search strategy is described in the Appendix S1. To identify relevant randomized trials, we also searched reference lists, related journals, clinical trial databases, and published guidelines.

### Study selection and outcomes

Three authors (TSH, LML, and CYL) independently screened the titles, abstracts, and full texts of trials identified by the literature search. We included randomized trials involving parenteral selenium supplementation that was administered in addition to routine nutritional interventions to adults (18 years or over) with critical illness. We did not include mixed immunonutrition or antioxidant interventions where selenium was one of several compounds administered. Due to concerns regarding pharmacokinetics, pharmacodynamics, and drug interactions that may affect the efficacy of enteral selenium supplementation, enteral selenium supplementation was not included. The primary outcome was all-cause mortality, and the secondary outcome was adverse events.

### Data extraction

We extracted the following information: characteristics of studies (publication year, study settings, designs, methods of randomization, and inclusion/exclusion criteria), characteristics of participants (age, sex, and disease), interventions (treatment strategy, dose, and duration), comparisons (types of control group), and outcomes (types of outcome measures, and adverse events). We retrieved data from individual studies based on the intention-to-treat principle. We extracted head-to-head comparison data for data synthesis where multiple arms were designed. For the primary outcome of all-cause mortality, we used 28-day mortality. If 28-day mortality was not reported, we used ICU mortality; if ICU mortality was not reported, we used hospital mortality. For adverse effects, we used definitions as defined in the included studies.

### Quality assessment

Two authors (CYL and LML) independently extracted data and assessed trial quality. A third author (TSH) was consulted to resolve disagreements and for quality assurance. We evaluated the methodological quality of the included trials using a domain-based evaluation that included the following risk of bias domains: selection bias (random sequence generation and allocation concealment), performance bias (blinding of participants and personnel), detection bias (blinding of outcome assessment), attrition bias (incomplete outcome data), and reporting bias (selective reporting) [21].

### Data synthesis and statistical analysis

We performed data synthesis on studies that enrolled critically ill patients with SIRS or sepsis. We analysed dichotomous outcomes extracted from individual studies to compute individual study relative risks (RRs) with 95% confidence intervals (CIs) and estimate the pooled Mantel-Haenszel (M-H) RR and the associated 95% CIs. We used a random-effects model (DerSimonian-Laird method) to estimate overall M-H RR due to pragmatic distributional treatment effects [22], [23]. We assessed clinical heterogeneity by comparing the protocols and methodologies of the included studies. Statistical heterogeneity of effect sizes between studies was assessed using the *I^2^* statistic and the Q statistic with the χ^2^ test [24], [25]. We defined statistical heterogeneity using a cut-off value of *p*≤0.10 for the χ^2^ test results or *I^2^*≥50%. We used Review Manager (version 5.1) for data synthesis and subgroup analyses. We explored study-level heterogeneity using pre-specified subgroup analyses (duration, administration strategies, and treatment dose) [26]–[28]. Univariate random-effects meta-regression was performed using R software (version 2.14.1) for continuous variables. In addition, we conducted sensitivity analysis according to different outcome definitions, types of control, and study quality to test the robustness of our results. We also constructed a funnel plot to evaluate publication bias. To calculate the number needed to treat (NNT), we used the formula NNT = 1/(absolute risk difference). Two-sided p-values ≤0.05 were considered statistically significant for hypothesis testing.

## Results

**Figure 1:**
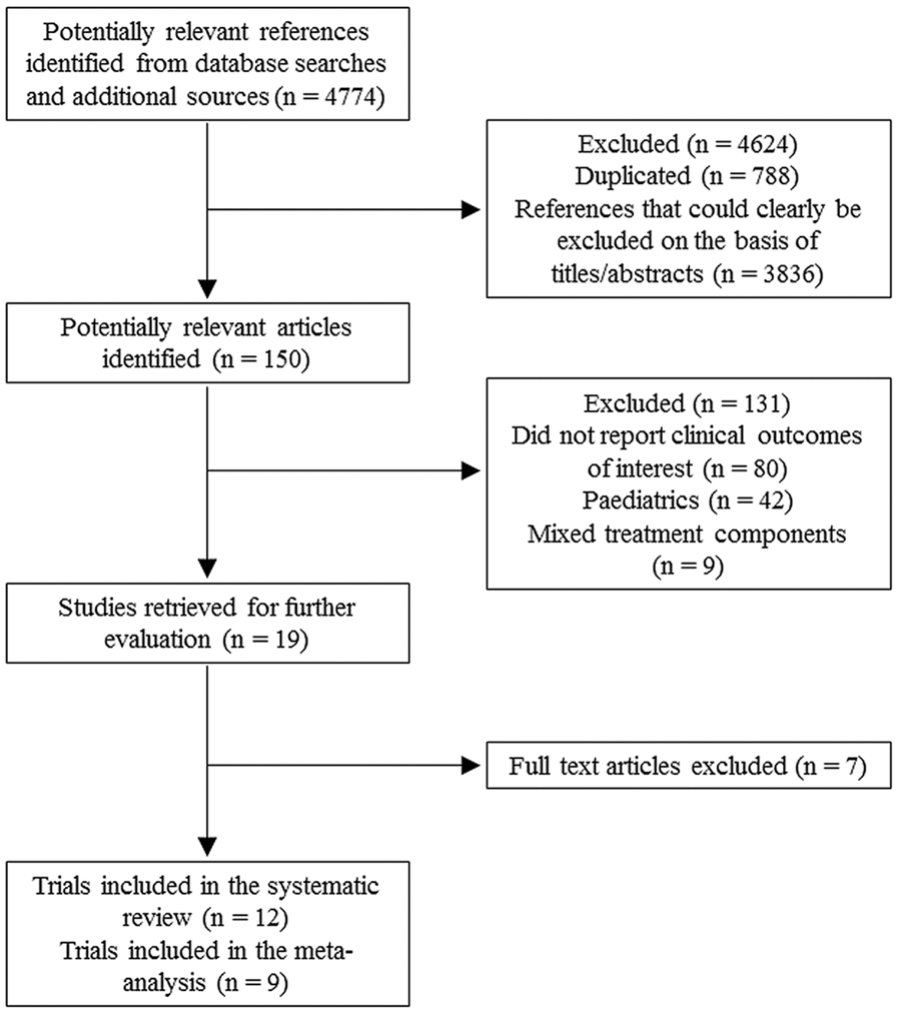
Flow diagram of the article-selection process.

Figure 1 summarizes the literature search results and identification of eligible studies. We excluded seven references after a comprehensive review of the full texts (Table S1). We finally included 12 randomized trials [17]–[19], [29]–[38]. Table 1 outlines the key characteristics of included trials. Most included trials were conducted in Europe, including five performed in Germany. Three articles [29], [30], [33] were in German and one was in Spanish [38]. Two enrolled only patients who had acute pancreatitis [29], [33]. One study recruited patients with multiple trauma [32]. Three included studies were multi-center trials [19], [34], [37].

**Table 1:** Studies included in the systematic review of the effects of supplemental parenteral selenium treatment in critically ill patients.

Author (Year) [Reference]	Location	Inclusion criteria	Treatment/Control	Treatment duration	No. of patients with positive cultures/total no. of patients (%)	No. of mortality in control group/total no. of control group (%)	Blind
Kuklinski et al (1991) [29]	One hospital, Germany	Contrast CT: Pancreatic necrosis, onset ≤72 hours	T: Se 500 µg/dayC: No treatment	Unknown	NA	8/9 (89)	No
Zimmermann et al (1997) [30]	One hospital, Germany	SIRS and organ failure	T: Se 1000 µg loading bolus, thereafter Se 1000 µg/day continuous infusion for 28 daysC: No treatment	28 days	40/40 (100)	8/20 (40)	No
Angstwurm et al (1999) [31]	One ICU, Germany	APACHE score ≥15, and clinical and laboratory signs of new SIRS according to sepsis criteria first 24 hours after admission	T: Se 535 µg/day for 3 days, 285 µg/day for 3 days, 155 µg/day for 3 days, 35 µg/day thereafterC: Placebo of saline and Se 35 µg/day	9 days	33/42 (79)	11/21 (52)	No
Berger et al (2001) [32]	One ICU, Switzerland	Severe multiple injury (ISS>15), requiring ICU support, age 18–75 years, admission within 24 hours of injury	T 1: Se 500 µg/dayC: VehicleT 2: Se 500 µg/day, zinc 13 mg/day, and 150 mg alpha-tocopherol in 5 mL 10% lipid emulsion once daily	5 days	NA	1/12 (8)	Double-blind
Lindner et al (2004) [33]	One centre, Germany	Severe acute pancreatitis, onset ≤72 hours	T: Se 2000 µg for 1 day, Se 1000 µg/day for 4 days, Se 300 µg/day until dischargedC: 0.9% sodium chloride placebo	Unknown	NA	3/35 (9)	No
Mishra et al (2007) [36]	One centre, ICU, UK	APACHE II score >15, clinical suspicion of infection and >1 organ dysfunction	T: Se 470 µg/day for 3 days, 320 µg/day for 3 days, 160 µg/day for 3 days, 30 µg/day thereafterC: Se 30 µg/day	9 days	37/40 (93)	11/22 (50)	Double-blind
Angstwurm et al (2007) [34]	Multiple centres, 11 ICUs, Germany	Age ≥18 years with APACHE III score >70 and SIRS; admission within 24 hours	T: 1000 µg loading bolus, followed by 1000 µg/day continuous infusion for 14 days. Allowed Se from other preparations up to 100 µg/day.C: Matching placebo of 0.9% sodium chloride. Allowed Se from other preparations up to 100 µg/day	14 days	199/238 (84)	61/124 (49)	Double-blind
Forceville et al (2007) [35], [37]	Seven centres, ICUs, France	Age ≥18 years, admission to ICUs and severe documented infection, severe septic shock, SAPS II ≥25	T: 4000 µg loading infusion for day 1, followed by 1000 µg/day continuous infusion for following 9 daysC: Placebo	10 days	40/40 (100)	13/29 (45)	Double-blind
Montoya et al (2009) [38]	One centre, ICU, Mexico	ICU admission with a diagnosis of sepsis, age >18 years	T: Se 1000 µg on the first day, 500 µg on the second day and 200 µg/day for following daysC: Se 100 µg/day	10 days	68/68 (100)	8/34 (24)	Double-blind
Andrews et al (2011) [19]	10 centres, ICUs, Scotland, UK	ICU admission ≥48 hours, age ≥16 years, and required ≥50% of their nutritional requirements to be met by parenteral nutrition	T 1: Se 500 µg/dayC: Se ≤50 µg/dayT 2: Glutamine+Se ≤50 µg/dayT 3: Glutamine+Se 500 µg/day	4.1 days	132/249 (53)	38/125 (30)	Double-blind
Valenta et al (2011) [17]	One centre, ICU, Czech Republic	Patients with SIRS/sepsis and SOFA score >5, age >18 years	T: 1000 µg Se loading bolus for 1st day, followed by 500 µg/day bolus administration for 14 days+standard Se doseC: Standard Se dose (<75 µg/day)	14 days	122/150 (81)	24/89 (27)	No
Manzanares et al (2011) [18]	One centre, ICU, Uruguay	SIRS patients, APACHE II score >15, predicted mechanical ventilation for >48 hours	T: 2000 µg Se loading bolus, followed by 1600 µg/day continuous infusion for 10 daysC: 0.9% sodium chloride	10 days	22/31 (71)	6/19 (32)	Single-blind

*ITT,* intention to treatment; *Se,* selenium; *ICU,* intensive care unit; *T,* treatment group; *C,* control group; *APACHE,* acute physiology and chronic health evaluation; *SOFA,* sequential organ failure assessment; *SIRS,* systemic inflammatory response syndrome; *ISS,* injury severity score; *SAPS,* Simplified Acute Physiologic Score; *CT,* computed tomography; *No.,* number; *NA,* not applicable.

Among 12 randomized trials, eight studies directly recruited patients with SIRS or sepsis [17], [18], [30], [31], [34]–[36], [38]. Patients with positive cultures ranged from 70% to 100% in these eight studies. The SIGNET trial [19] (Scottish Intensive care Glutamine or seleNium Evaluative Trial) included patients with admission to ICU for more than 48 hours, and required more than 50% of nutritional requirements to be met by parenteral nutrition. Fifty-three percent of participants enrolled in this study had sepsis. We conducted data synthesis and meta-analysis of these nine trials. Five of these nine randomized trials were double-blinded. These nine trials comprised 965 participants, of whom 482 were randomized to parenteral selenium supplementation and 483 to control. Among patients assigned to treatment groups, 148 (30.7%) died, while 180 patients (37.3%) assigned to the control groups died. Mortality rates among control groups ranged from 24% to 52%.

Administration schedules (durations, strategies, and doses) varied considerably between studies. The current recommendation for selenium intake in humans is 55 to 75 µg per day [10], [39]. Therefore, we calculated therapeutic duration (days) by durations of intervention arms treated with more than 100 µg selenium. We defined the loading bolus group based on whether the loading dose given by a bolus administration on the first day. Three studies administered loading bolus followed by continuous infusion [18], [30], [34] and one study employed only loading bolus administration [17]. To define the high dose group, we adopted the 1000 µg per day as the cut-off point throughout the therapeutic periods and four studies were included [18], [30], [34], [35]. Controls included no treatment, placebo, or maintenance dose. Control arm maintenance doses were all ≤100 µg per day. The SIGNET trial had a 2×2 factorial design. Thus, data were extracted from the parenteral selenium monotherapy arm and control arm for data synthesis.

A summary of the risk of bias is provided (Table S2). The included studies varied in the reporting of random sequence generation and allocation concealment. Blinding of participants and personnel was adequate in five studies. All studies were low risk for detection bias due to the hard outcome, mortality. Most of included studies were low risk for attrition bias.

**Figure 2:**
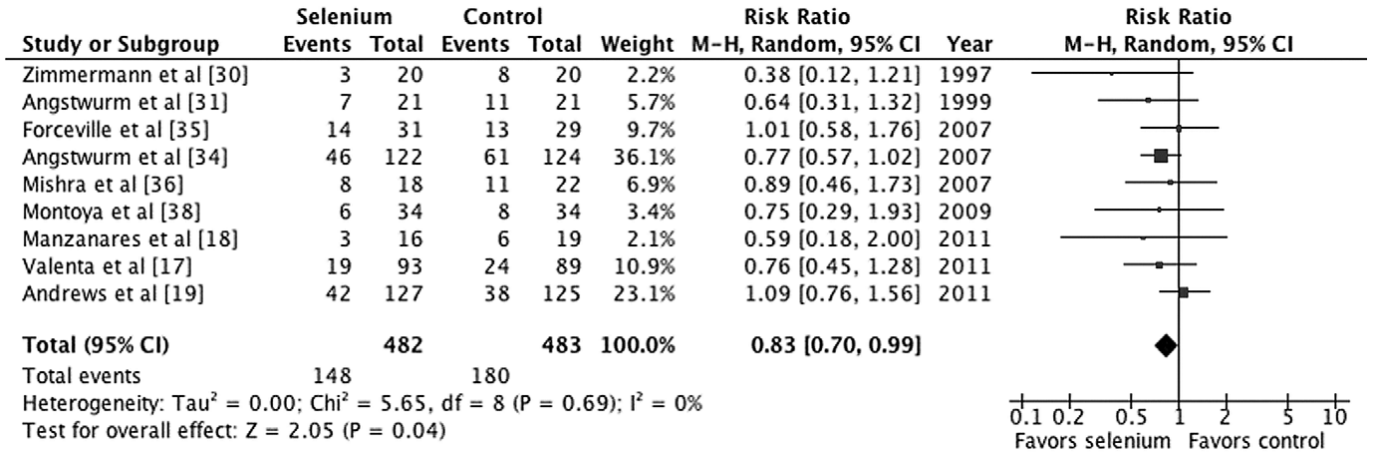
Forest plot comparing mortality among selenium-treated patients to that of controls among critically ill patients with systemic inflammatory response syndrome (SIRS) or sepsis.

**Figure 3:**
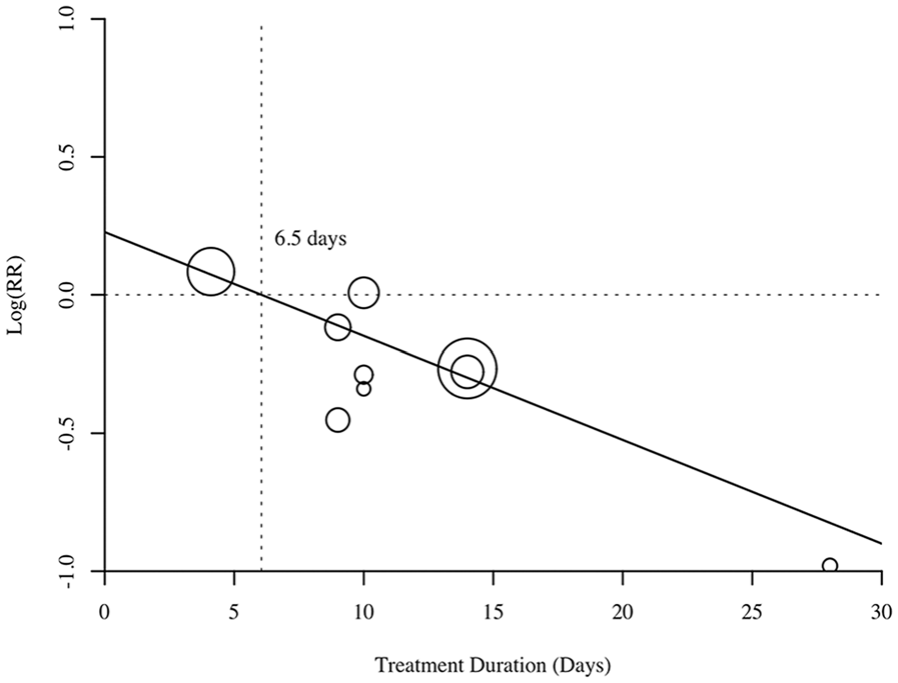
Meta-regression analysis of selenium treatment duration on log relative risk. Each circle represents a trial study. The size of each circle is proportional to the weight assigned to the corresponding study. The majority of trials with longer duration (>7 days) have negative effect sizes. A negative effect size implies a reduction in mortality rate.

Overall, the effect of parenteral selenium supplementation on all-cause mortality was statistically significant (M-H RR 0.83, 95% CI 0.70–0.99, *p* = 0.04; Figure 2). There was little evidence of between-study heterogeneity (*I^2^* = 0%, *p* = 0.69). For an assumed control event rate of 37.3% (derived from the pooled estimate of the control groups), NNT was 16 (95% CI 9–271). To investigate the effect of treatment duration on study-level estimates of the relative risk of mortality, we performed a univariate random-effects meta-regression analysis. Meta-regression revealed a statistically significant association between the log RR and duration of treatment (coefficient, −0.037; standard error, 0.019; *p* = 0.047; Figure 3).

**Figure 4:**
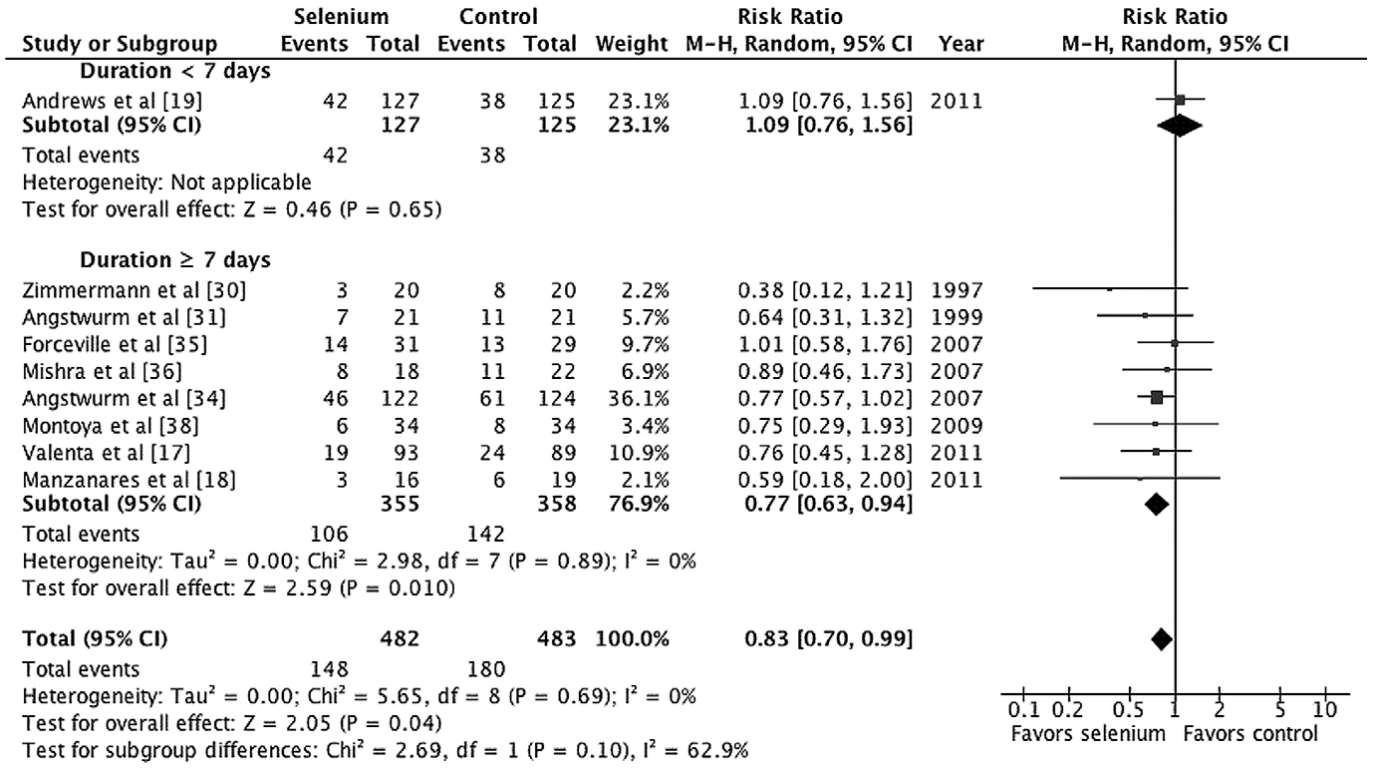
Forest plot comparing mortality of selenium-treated patients to controls by treatment durations.

**Figure 5:**
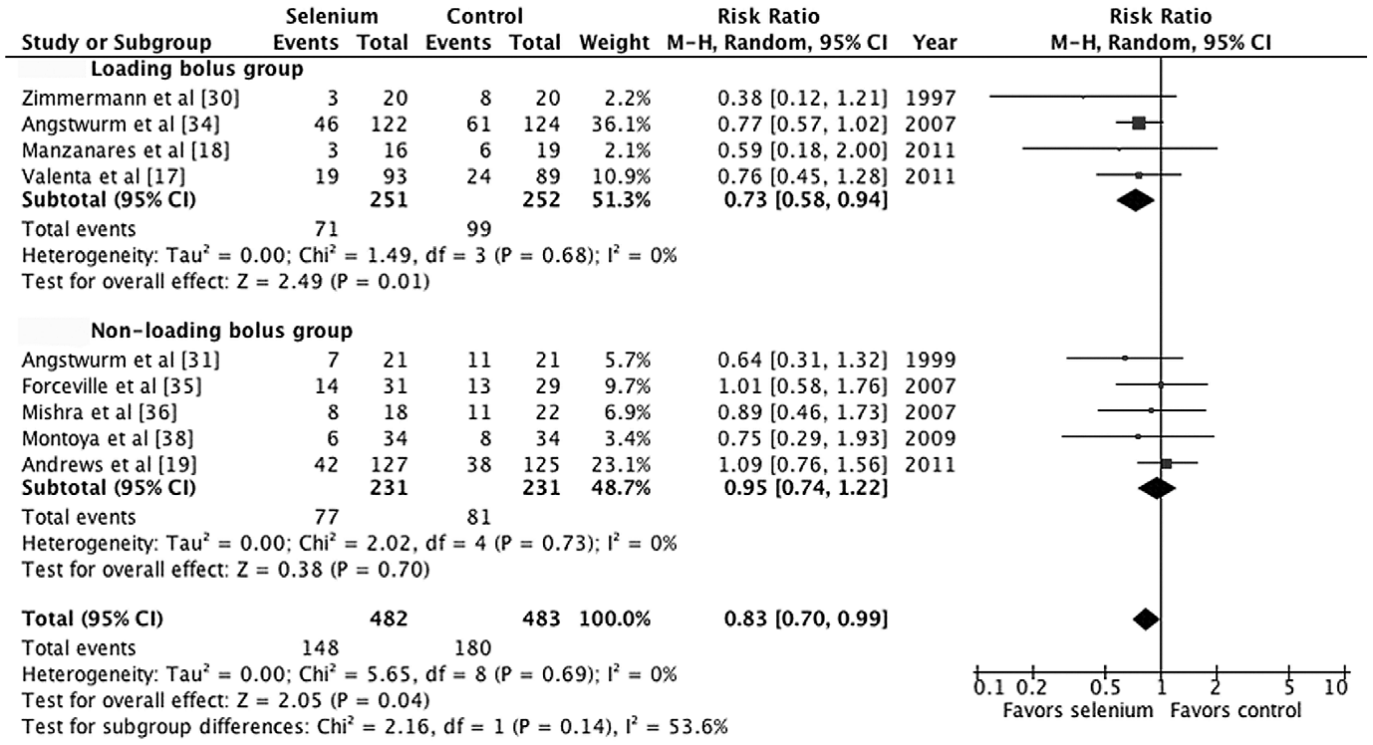
Forest plot comparing mortality among selenium-treated patients to controls by administration strategies.

**Figure 6:**
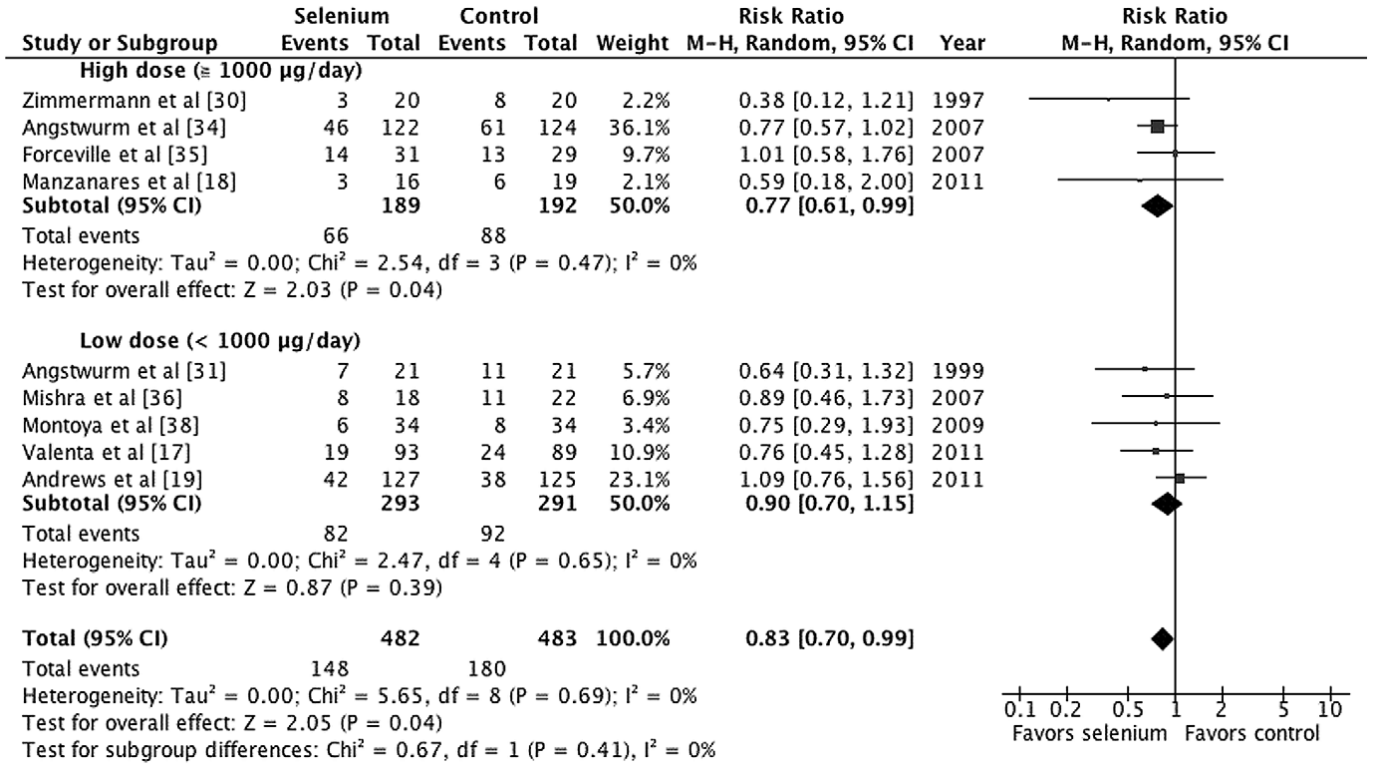
Forest plot comparing mortality among selenium-treated patients to controls by treatment dosages.

We conducted subgroup analyses based on administration durations, strategies, and doses. Treatment duration of 7 days was the cut-off point based on the results of the meta-regression and the pharmacokinetics study [17], [18], [40]. Subgroup analysis demonstrated substantial heterogeneity between subgroups for treatment duration as a covariate; however, there was not statistically significant (*p* = 0.10, *I^2^* = 62.9%; Figure 4). Parenteral selenium supplementation that lasted at least 7 days was associated with a statistically significant reduction in mortality (M-H RR 0.77, 95% CI 0.63–0.94, *p* = 0.01). For an assumed control event rate of 37.3%, NNT was 12 (95% CI 8–45). The subgroup analysis based on administration strategies shows that the loading bolus group has lower mortality rate than non-loading bolus group but the difference was not statistically significant (*p* = 0.14, *I^2^* = 53.6%, Figure 5). Parental selenium supplementation with loading bolus was associated with a statistically significant reduction in mortality (M-H RR 0.73, 95% CI 0.58–0.94, *p* = 0.01). Furthermore, there was a lower mortality risk among patients who received high-dose selenium treatment compared with control than those who received low-dose selenium treatment compared with control, but the subgroup differences were not statistically significant (*p* = 0.41, *I^2^* = 0%, Figure 6).

Six trials reported a wide variety of adverse events. Manzanares and colleagues [18] reported no adverse events in either group. We pooled the adverse events of the other five trials and compared the adverse events that occurred among selenium-treated patients to those that occurred among controls. There was no evidence that selenium supplementation was harmful (M-H RR 1.01, 95% CI 0.76–1.33, *p* = 0.97, *I* ^2^ = 47%; Figure S1). Inconsistent definitions on adverse effects across studies might have been the cause of the heterogeneity.

To test the robustness of our results, we conducted sensitivity analysis according to the different definitions of outcome measures (28-day mortality, ICU mortality, and hospital mortality), types of control, and risk of bias (performance bias and attrition bias). There was no statistical significance of subgroup differences for different definitions of outcome measures, types of control, performance bias, and attrition bias (Table S3). The funnel plot was symmetric for large studies (Figure S2). Small studies with negative effects might be missing, and if they are, the true effect might be smaller than the observed effect.

## Discussion

This meta-analysis provides evidence that parenteral selenium supplementation significantly reduces all-cause mortality in critically ill patients with SIRS or sepsis. Administration of parenteral selenium treatment to 16 critically ill septic patients will prevent one additional death. The treatment strategy employing the loading dose given as a bolus administration with longer treatment duration might further reduce all-cause mortality in septic patients. However, this finding should be taken cautiously because all loading bolus trials from our collected trials also have longer durations. It is not possible to determine whether one factor is sufficient or both factors are required for better performance of parenteral selenium supplementation based on our results.

Recent advances in understanding the pathophysiology of sepsis further support the results of our meta-regression and subgroup analysis. Immune response during sepsis is an exuberant inflammatory reaction. The proposed immunopathology of sepsis indicates that pro-inflammatory responses predominate in the early phase, with a shift to anti-inflammatory responses through complex mechanisms as sepsis progresses [41]. Prompt treatment with antibiotics and evidence-based supportive care has enabled the majority of patients to survive the pro-inflammatory phase and enter a protracted immunosuppressive stage [5], [41]. During the protracted immunosuppressive stage, most deaths occur as a result of nosocomial infection with virulent pathogens or reactivation of latent viruses [42]–[44]. Methods to reverse or prevent this immune deficiency and enhance patient recovery need to be a major focus of future research [4].

The optimal administration schedule of selenium treatment remains controversial [40]. A loading bolus given in the early phase of sepsis has been postulated to have several effects including downregulating the synthesis of proinflammatory cytokines, induction of apoptosis and cytotoxicity in activated proinflammatory circulating cells, and direct virucidal and bactericidal effects [45]. As to treatment strategies, Manzanares and Hardy [40] revealed that both high-dose (2000 µg loading and 1600 µg/day) and low-dose (1200 µg loading and 800 µg/day) of parenteral selenium supplementation restored selenium deficiency in septic patients. GPx-3 activity increased with both doses, but only maintained normal physiological ranges in patients with SIRS who were treated with high-dose selenium after day 7. However, Valenta and colleagues revealed that parenteral selenium supplementation employing only boluses given with 1000 µg selenium on day one followed by 500 µg per day for 14 days restored selenium deficiency and GPx-3 activity in septic patients [17]. Thus, administration strategies (a loading bolus followed by continuous infusion or a loading bolus followed by boluses), and optimal treatment dose deserves further future evaluation.

Our meta-analysis constitutes up-to-date evidence for parenteral selenium supplementation in critically ill patients with SIRS or sepsis. In 2008, Avenell and colleagues [16] updated their meta-analysis of selenium supplementation in critically ill adults in The Cochrane Library. They concluded that there was little evidence to recommend selenium supplementation in critically ill adults. In the meta-analysis recently published by Manzanares and colleagues [46], it was demonstrated that supplementation with antioxidants including trace elements, vitamins, and glutamine may reduce the hospital mortality in critically ill patients, particularly in the high-risk group (>10% mortality in the control group). Their meta-analysis pooled 20 randomized trials of mixed parenteral and enteral antioxidant therapies in critically ill patients within heterogeneous intensive care populations. The result of their meta-analysis of parenteral selenium monotherapy was not statistically significant (RR 0.86, 95% CI 0.73 to 1.01, *p* = 0.06). Based on these results, whether combination therapy with different pharmaconutrients such as amino acids, nucleotides, omega-3 polyunsaturated fatty acids, vitamins, and other trace elements results in synergistic effects should be further investigated.

Our meta-analysis has several limitations. First is the variation in the methodological quality of the included studies, which resulted from heterogeneous reports and study designs. Although we could not exclude performance bias (the χ^2^ test is often low power), we believe that the degree of overestimation of the effect size is mild. Second, subgroup analyses and meta-regression analyses describe observational relationships across studies. We conducted subgroup analyses and meta-regression based on pre-specified variables in our published protocol to avoid spurious findings. Moreover, plausible biological and pharmacological mechanisms and similar findings from other studies are of crucial importance in interpreting the results of these analyses [28]. Finally, regarding publication bias, the true effect might be smaller than the observed effect if small negative effect trials were missing.

## Conclusions

Parenteral selenium treatment significantly reduces risk of mortality among critically ill patients with SIRS or sepsis without evident adverse effects. Based on our results, guidelines making recommendations on parenteral selenium supplementation for critically ill septic patients should be considered. Owing to the varied methodological quality of the studies, future high-quality randomized trials that directly focus on the effect of adequate-duration of parenteral selenium supplementation for severe septic patients are needed to confirm our results. Treatment strategies may consider including a loading dose given as a bolus. Incorporation of biomarkers to optimize treatment effects should be considered. Future clinical trials in sepsis research should consider adequate treatment duration when designing treatment periods.

## Supporting Information

Figure S1 **Forest plot comparing adverse events in treatment and control groups.**
(TIF)

Table S3 **Relative risk of mortality comparing parenteral selenium supplementation with control, from subgroup analysis.**
(DOC)

Figure S2 **Funnel plot of the standard error by log relative risk of mortality.**
(TIF)

Table S1 **Studies excluded from the meta-analysis of randomized trials involving parenteral selenium treatment of critically ill patients.**
(DOC)

Table S2 **Risk of bias summary: Review authors**' **judgements about each risk of bias item across all included studies.**
(DOC)

Appendix S1 **Search strategy (MEDLINE/OvidSP).**
(DOC)
