# Supplementary figures and images for: Effect of Parenteral Selenium Supplementation in Critically Ill Patients: A Systematic Review and Meta-Analysis
Source: PLoS One. 2013 Jan 25;8(1):e54431. doi: 10.1371/journal.pone.0054431 (PMC3555933; doi:10.1371/journal.pone.0054431)

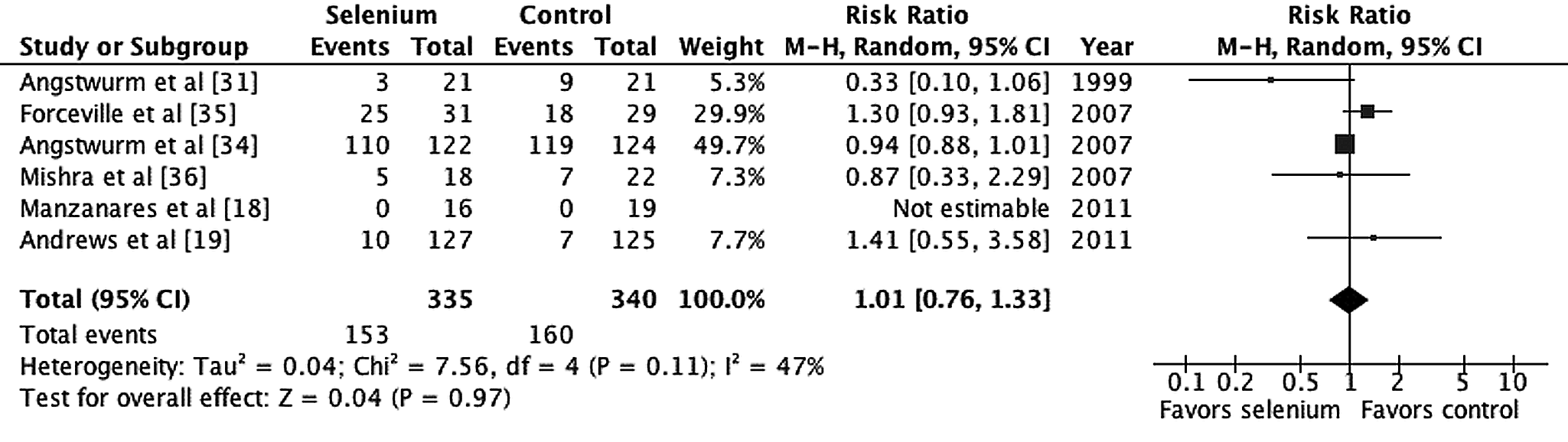

Supplement: Figure S1 — Forest plot comparing adverse events in treatment and control groups. (TIF) [file pone.0054431.s001.tif]
