# Supplementary material for: Effect of Parenteral Selenium Supplementation in Critically Ill Patients: A Systematic Review and Meta-Analysis
Source: PLoS One. 2013 Jan 25;8(1):e54431. doi: 10.1371/journal.pone.0054431 (PMC3555933; doi:10.1371/journal.pone.0054431)
Supplement: Table S3 — Relative risk of mortality comparing parenteral selenium supplementation with control, from subgroup analysis. (DOC) [file pone.0054431.s002.doc]

**Table S3.**

**Relative risk of mortality comparing parenteral selenium supplementation with control, from subgroup analysis.**

|  | **M-H RR (95% CI)** | **Test for subgroup differences** |
| --- | --- | --- |
| **Types of control** |  |  |
| Maintenance dose ≤100 μg/day | 0.84 (0.70–1.01) | p=0.38, *I^2^*=0% |
| Placebo | 0.92 (0.55–1.53) |  |
| No treatment | 0.38 (0.12–1.21) |  |
| **Types of outcome definition** |  |  |
| 28-day mortality | 0.79 (0.64–0.98) | p=0.42, *I^2^*=0% |
| ICU mortality | 1.04 (0.73–1.47) |  |
| Hospital mortality | 0.81 (0.58–1.13) |  |
| **Performance bias** |  |  |
| High risk | 0.65 (0.45–0.96) | p=0.16, *I^2^*=48.6% |
| Low risk | 0.89 (0.73–1.08) |  |
| **Attrition bias** |  |  |
| High risk | 0.76 (0.59–0.99) | p=0.36, *I^2^*=0% |
| Low risk | 0.90 (0.71–1.14) |  |

*M-H,* Mantel-Haenszel; *ICU,* intensive care units
