# Supplementary material for: Effect of Parenteral Selenium Supplementation in Critically Ill Patients: A Systematic Review and Meta-Analysis
Source: PLoS One. 2013 Jan 25;8(1):e54431. doi: 10.1371/journal.pone.0054431 (PMC3555933; doi:10.1371/journal.pone.0054431)
Supplement: Table S1 — Studies excluded from the meta-analysis of randomized trials involving parenteral selenium treatment of critically ill patients. (DOC) [file pone.0054431.s004.doc]

**Table S1.**

**Studies excluded from the meta-analysis of randomised trials involving** **parenteral selenium treatment of critically ill patients.**

| **Study** | **Reason for exclusion** |
| --- | --- |
| Yamaguchi [1] | RCT in adults with acute ischemic stroke. Selenium administered orally. |
| Saito [2] | RCT in adults with aneurysmal subarachnoid haemorrhages. Selenium administered orally. |
| Bertolini [3] | RCT in adults, needed ventilation and nutrition for at least 4 days, compared enteral feeding with either an immune-enhancing formula or parenteral nutrition. |
| Angstwurm [4] | No clinical outcomes reported. |
| Berger [5] | RCT in adults with major burns. Mixed treatment: selenium, zinc, and copper. |
| Berger [6] | RCT in adults with organ failure after complicated cardiac surgery, major trauma, or subarachnoid haemorrhage. Mixed treatment: selenium, zinc, vitamin C, and vitamin B1. |
| El-Attar [7] | RCT in adults with COPD in RICU. Mixed treatment: selenium, zinc, and manganese. |

*RCT,* randomised controlled trials; *COPD,* chronic obstructive pulmonary disease; *RICU,* respiratory intensive care unit.

**References**

1. Yamaguchi T, Sano K, Takakura K, Saito I, Shinohara Y, et al. (1998) Ebselen in acute ischemic stroke: a

placebo-controlled, double-blind clinical trial. Ebselen Study Group. Stroke 29: 12-17.

2. Saito I, Asano T, Sano K, Takakura K, Abe H, et al. (1998) Neuroprotective effect of an antioxidant, ebselen,

in patients with delayed neurological deficits after aneurysmal subarachnoid hemorrhage. Neurosurgery 42:

269-277; discussion 277-268.

3. Bertolini G, Iapichino G, Radrizzani D, Facchini R, Simini B, et al. (2003) Early enteral immunonutrition in

patients with severe sepsis: results of an interim analysis of a randomized multicentre clinical trial. Intensive

Care Med 29: 834-840.

4. Angstwurm MW, Schopohl J, Gaertner R (2004) Selenium substitution has no direct effect on thyroid

hormone metabolism in critically ill patients. Eur J Endocrinol 151: 47-54.

5. Berger MM, Eggimann P, Heyland DK, Chiolero RL, Revelly JP, et al. (2006) Reduction of nosocomial

pneumonia after major burns by trace element supplementation: aggregation of two randomised trials. Crit

Care 10: R153.

6. Berger MM, Soguel L, Shenkin A, Revelly JP, Pinget C, et al. (2008) Influence of early antioxidant

supplements on clinical evolution and organ function in critically ill cardiac surgery, major trauma, and

subarachnoid hemorrhage patients. Crit Care 12: R101.

7. El-Attar M, Said M, El-Assal G, Sabry NA, Omar E, et al. (2009) Serum trace element levels in COPD

patient: the relation between trace element supplementation and period of mechanical ventilation in a

randomized controlled trial. Respirology 14: 1180-1187.
