# Supplementary material for: Effect of Parenteral Selenium Supplementation in Critically Ill Patients: A Systematic Review and Meta-Analysis
Source: PLoS One. 2013 Jan 25;8(1):e54431. doi: 10.1371/journal.pone.0054431 (PMC3555933; doi:10.1371/journal.pone.0054431)
Supplement: Table S2 — Risk of bias summary: Review authors' judgements about each risk of bias item across all included studies. (DOC) [file pone.0054431.s005.doc]

**Table S2.**

**Risk of bias summary: Review authors’ judgements about each risk of bias item across all included studies.**

| **Study** | **Random sequence generation (selection bias)** | **Allocation concealment (selection bias)** | **Blinding of participants and personnel (performance bias)** | **Blinding of outcome assessment (detection bias)** | **Incomplete outcome data (attrition bias)** | **Selective reporting (reporting bias)** |
| --- | --- | --- | --- | --- | --- | --- |
| Zimmermann et al [30] | Unclear | Unclear | High risk | Low risk | Low risk | Unclear |
| Angstwurm et al [31] | Unclear | Unclear | High risk | Low risk | Low risk | Low risk |
| Mishra  et al [36] | Unclear | Unclear | Low risk | Low risk | Low risk | Unclear |
| Angstwurm et al [34] | Unclear | Unclear | Low risk | Low risk | High risk | Unclear |
| Forceville  et al [35, 37] | Unclear | Unclear | Low risk | Low risk | Low risk | Low risk |
| Montoya  et al [38] | Unclear | Low risk | Low risk | Low risk | Low risk | Unclear |
| Andrews  et al [19] | Low risk | Low risk | Low risk | Low risk | Low risk | Low risk |
| Valenta  et al [17] | Unclear | Unclear | High risk | Low risk | High risk | Unclear |
| Manzanares et al [18] | Unclear | Unclear | High risk | Low risk | Low risk | Low risk |
