# Supplementary material for: Effect of Parenteral Selenium Supplementation in Critically Ill Patients: A Systematic Review and Meta-Analysis
Source: PLoS One. 2013 Jan 25;8(1):e54431. doi: 10.1371/journal.pone.0054431 (PMC3555933; doi:10.1371/journal.pone.0054431)
Supplement: Appendix S1 — Search strategy (MEDLINE/OvidSP). (DOC) [file pone.0054431.s006.doc]

**Appendix S1.**

**Search strategy (MEDLINE/OvidSP).**

1. selenium.mp. or exp Selenium Compounds/or exp Selenium/

2. selen*.mp. [mp=protocol supplementary concept, rare disease supplementary concept, title, original title, abstract, name of substance word, subject heading word, unique identifier]

3. 1 or 2

4. randomized controlled trial.pt.

5. controlled clinical trial.pt.

6. randomized.ab.

7. placebo.ab.

8. drug therapy.fs.

9. randomly.ab.

10. trial.ab.

11. groups.ab.

12. 4 or 5 or 6 or 7 or 8 or 9 or 10 or 11

13. exp animals/not humans.sh.

14. 12 not 13

15. 3 and 14
